# Association between body weight, physical activity and food choices among metropolitan transit workers

**DOI:** 10.1186/1479-5868-4-52

**Published:** 2007-11-02

**Authors:** Simone A French, Lisa J Harnack, Traci L Toomey, Peter J Hannan

**Affiliations:** 1 University of Minnesota, Division of Epidemiology & Community Health, Suite 300 WBOB, 1300 South Second Street, Minneapolis MN 55454

## Abstract

**Background:**

Associations between body weight, physical activity and dietary intake among a population of metropolitan transit workers are described.

**Methods:**

Data were collected during October through December, 2005, as part of the baseline measures for a worksite weight gain prevention intervention in four metro transit bus garages. All garage employees were invited to complete behavioral surveys that assessed food choices and physical activity, and weight and height were directly measured. Seventy-eight percent (N = 1092) of all employees participated.

**Results:**

The prevalence of obesity (BMI >= 30 kg/m^2^) was 56%. Over half of the transit workers reported consuming fruit (55%) and vegetables (59%) ≥ 3/week. Reported fast food restaurant frequency was low (13% visited ≥ 3/week). Drivers reported high levels of physical activity (eg. walking 93 minutes/day). However, an objective measure of physical activity measured only 16 minutes moderate/vigorous per day. Compared to other drivers, obese drivers reported significantly less vigorous physical activity, more time sitting, and more time watching television. Healthy eating, physical activity and weight management were perceived to be difficult at the worksite, particularly among obese transit workers, and perceived social support for these behaviors was modest. However, most workers perceived weight management and increased physical activity to be personally important for their health.

**Conclusion:**

Although transit workers' self-report of fruit and vegetable intake, and physical activity was high, perceived access to physical activity and healthful eating opportunities at the worksite was low. Obese workers were significantly less physically active and were more likely to report work environmental barriers to physical activity.

## Background

### Overweight and obesity are major public health problems

Overweight and obesity are increasing in the US population at an alarming rate. During the past ten years, the prevalence of obesity increased by 33%, from 22.9% in 1988 to 30.5% in 1999/2000 [1]. Currently, 69% of US adults are overweight or obese [2]. Overweight and obesity are a major public health concern because of their high prevalence in the population and their link with serious health morbidities such as hypertension, Type 2 diabetes, hypercholesterolemia, cardiovascular disease, and some cancers. Weight gain during adulthood increases disease risk, independent of initial body weight [3]. On average, US adults gain about 1–2 lbs per year [3].

### Transportation workers are at high risk for overweight and obesity

In comparison with people in other occupations, transportation workers are at higher risk with respect to obesity, sedentary behaviors, and dietary intake [4-6]. The empirical literature consistently shows higher rates of mortality, morbidity, and absence due to illness among bus drivers, compared with other occupational groups [4]. Both individual-level behaviors and work environmental variables probably contribute to higher obesity prevalence and risk of excess weight gain in this occupational group [4,6].

### Worksite environment is an important influence on food and physical activity behavior

Environmental influences are widely recognized as important contributors to excess weight gain and the development of overweight and obesity [7,8]. The majority of the adult population is exposed to the worksite environment for a significant portion of their day over a period of years [9]. Worksite physical environments provide opportunities and exposures that influence individual food choices and physical activity behaviors [7]. The social environment at the worksite is also an important worksite environmental influence on individual food and physical activity behaviors.

Long work hours, shift work, lack of scheduled breaks or meals, and lack of healthful food and physical activity options on the transportation routes or in the transportation hubs (eg. bus or train garage) are some of the structural variables that make healthful food choices and physical activity behaviors difficult for transportation workers [5]. While some of the structural work variables involve policy changes at the transit system level (eg. bus driver schedules), others are modifiable at the garage level and could be addressed in a worksite intervention (eg. availability of healthful food choices in vending machines; availability of fitness equipment at garages) [5,7].

The present paper reports baseline data from a worksite obesity prevention program that targeted bus drivers at four bus garages in a metropolitan area. The study was one of several funded by the National Institutes of Health to examine worksite obesity prevention interventions. The purpose of the present paper is to describe the prevalence of overweight and obesity, and its association with food choices and physical activity behaviors, among this unique group of workers who are at high risk for excess weight gain and obesity. The broader aim is to better understand how the work environment and individual behavioral variables may contribute to obesity and excess weight gain in this high-risk group of workers. This information may be useful in the design of effective obesity prevention interventions that target the worksite food and physical activity environment.

## Methods

### Study population and recruitment

The Route H study was conducted in collaboration with the Metro Transit Council of Minneapolis, Minnesota. Five garages in the metropolitan Minneapolis-St Paul area were selected to take part in the study. These five garages comprised all of the garages available in the metro area. Four garages (two urban; two suburban) were randomized to intervention or control. The fifth garage was selected to serve as the pilot garage. This was because it was very different from the other two garages. The pilot garage was much newer, with new exercise facilities and locker rooms on site. The break room and commons area were very spacious and modern. The facility was much larger than the other four garages. Garage was the unit of randomization, intervention and evaluation. The four garages were paired on physical characteristics and then randomized in pairs to intervention or comparison conditions by the toss of a coin. Due to the intervention timeline and the need to collect key interviews in intervention garages for intervention development, garages were randomized about six months prior to the start of the baseline data collection. The study obtained approval from the University of Minnesota IRB Human Subjects Protection Program.

Participants in the baseline data collection were recruited using a variety of methods, including paycheck distribution fliers, signs posted in the garages, fliers distributed at health fair events, information in employee newsletters and instant text messaging on the buses. All garage employees who worked at each of the four garages were eligible to complete the evaluation measures. Participants received a $20 incentive for completing the behavioral measurement survey and for having their height and weight measured by trained research staff.

In addition to the individual incentives, garage-level incentives were offered. Garages were offered a financial incentive that increased with the participation rate for the garage. Garages that attained a 60–69% garage employee participation rate received $2,500; a 70–79% participation rate earned $3,000; an 80–89% participation rate earned $5,000; and a 90% or higher participation rate earned $6,000. The average individual participation rate in the garages was 78% (n = 1121/1441 eligible completed the questionnaire at minimum). Complete height, weight and questionnaire data were completed by 1092 individuals. The four garage-level participation rates were: Intervention garages: 81.4%; 83.8%; Control garages: 82.5% and 69.2%. The lower participation rate in one of the control garages was probably due to this garage being the largest in workforce size, and in having a large proportion of the drivers that pick up their bus route in the field, not at the garage. This meant that drivers did not routinely stop in at the garage, and therefore were less directly exposed to the study staff when they were conducting measurement sessions at the garage.

Data collection sessions were held at various times and dates in each of the four garages. Bus drivers and other garage employees could complete the measures on site without a prior appointment. The survey and weight and height measures took about 45 minutes to complete. A selected subset of employees who participated in the main survey at each garage were invited to take part in one of two additional data collection sub-studies: a single telephone-administered 24-hour dietary recall (n = 158) or a four-day accelerometry measurement (n = 158). All employees were randomly assigned prior to the initiation of the primary data collection sessions to receive an invitation to participate in either the 24-hour dietary recall or the four-day accelerometry sub-study. An additional incentive of $10 was offered to participants for completing the dietary recall measurement. An additional $20 was offered to participants who wore an accelerometer for four days.

When 40 participants had completed each of the substudies at each garage, no more employees were invited to participate in the substudies. Substudy participation rates were difficult to compute due to the logistics of recruitment. For example, because only 12 accelerometers were available, once these were in use, recruitment for the accelerometer substudy was paused until more accelerometers were available. This meant that workers who were eligible for the accelerometer substudy were not invited to participate if they completed the main measurements during a period in which no accelerometers were available. Similarly, a pause in recruitment was implemented when the Nutrition Coordinating Center staff conducting the telephone recalls became back-logged due to heavy enrollment periods in the dietary recall substudy. These patterns of recruitment and enrollment in the substudies were unavoidable given the constraints of the data collection logistics at the garages. Participation rates were calculated based on the number of people who were invited to participate and agreed. The participation rate for the accelerometry substudy was 68% and for the dietary recall substudy was 81%. For the accelerometry study, the age and body mass index were almost identical among those who participated and the larger transit worker population who took part in the main survey. However, a slightly greater proportion of women completed the accelerometry study than were present in the larger survey sample (30% women in the accelerometry substudy versus 20% in the larger survey sample; 47 women of 158 total in the substudy sample). For the dietary recall, the body mass index and gender distributions were virtually identical among the substudy sample and the larger survey sample (33 women of 158 total). However, dietary recall substudy participants were slightly older than the larger survey sample participants (49.1 years versus 47.4 years, respectively).

## Measures

### Weight and height

Body weight was measured in street clothing without shoes using a calibrated electronic scale. Height was measured using a portable stadiometer. Two separate measurements were conducted for both the weight and the height measures. The average of the two values were used in statistical analyses. Trained and certified research staff conducted all anthropometric measurements. Body mass index was calculated as weight (kg)/height (m^2^). Obesity was defined as BMI >= 30 kg/m^2^; overweight was defined as BMI >= 25 kg/m^2 ^< 30 kg/m^2^. Normal weight was defined as BMI < 25 kg/m^2^.

### Dietary intake

Dietary intake was measured in two ways. Among a subset of 40 bus drivers at each of the four garages, a single, telephone-administered 24-hour dietary recall was collected. Recalls were collected as close as possible to the baseline data collection session at which the participant was recruited to complete the dietary recall substudy. However, there were significant challenges reaching bus drivers to conduct the telephone-based dietary recall. The University of Minnesota Nutrition Coordinating Center conducted the single unannounced dietary recalls using a multiple-pass procedure by staff trained and certified in the use of the Nutrition Data Systems for Research (NDS-R) dietary assessment software and protocol [10-14]. The multiple pass procedure consists of asking the participant to make an initial list of all of the foods and beverages consumed during the previous 24 hour period (first pass). This list is then reviewed to allow for corrections and additions (second pass). More detailed probing follows to elicit information on portion sizes and preparation methods (third pass). The final pass (fourth pass) involves reviewing the information collected to allow the participant a final opportunity to make corrections or additions. A single dietary recall produces valid estimates of macronutrient intake for group-level data such as that in the present study [15,16]. Total energy, percent fat energy, and servings per day of specific foods and beverages targeted by the intervention were estimated (eg. fruits, vegetables, sugar-sweetened beverages, snack foods, sweets/candy). The percent of drivers who met the fruit and vegetable recommendation of five or more servings per day was calculated by summing fruit and vegetable servings per day.

A second measure of food intake was collected from all participants using a self-report food frequency questionnaire. The instrument was adapted from two existing instruments for which validity has been evaluated [17,18]. Participants reported their weekly frequency during the past month of consumption of foods targeted by the intervention, such as fruits and vegetables (3 items), high fat snack foods (9 items), sugar sweetened beverages (2 items), and fast food restaurant use (1 item). Response options were "1–3 times last month", "1–2 times per week", "3–4 times per week", "5–6 times per week", and "7 or more times per week". Summary scores were computed for the fruit and vegetable items, high fat snack food items and sugar sweetened beverage items. Scores were calculated by multiplying the frequency of consumption per week times the usual portion size and summing across items. Due to the differences in portion size quantification among items, it is not possible to specify a particular unit for the score. Higher scores reflect consumption of larger amounts of the items included in the scale.

### Physical activity

Physical activity was measured with three different instruments. Among a subset of 40 bus drivers at each of the four garages, physical activity data during four consecutive days was measured using an Actigraph accelerometer [19-22]. The Actigraph has been shown to be a reliable and valid measure of physical activity in adults. Participants were instructed to wear the Actigraph for four consecutive days. The average daily number of minutes of strenuous, moderate and mild activity were calculated for each participant by summing the daily number of vector magnitude readings greater than 2100 counts/minute for strenuous/moderate activity, 251–2100 counts/minute for light, and 1–250 counts per minute for inactivity. Days in which there were more than 16 hours of consecutive zero readings were dropped from the analysis, and only three days of data were used to calculate averages for that person. Only 15 people had only three days of data.

A second method used to estimate physical activity was by self-report among all participants using a modified version of the International Physical Activity Questionnaire (IPAQ) [23-26]. Based on our developmental work with a pilot garage and its employees, modifications were made to the IPAQ to shorten the instructions and to simplify response formats. Participants reported the minutes per day during a seven-day period that they spent walking, sitting, in vigorous and in moderate physical activities across work, home and leisure settings. Minutes were summed across settings to provide a total minutes score for each of the variables walking, sitting, moderate and vigorous activity. The percent of drivers who met the recommended level of 150 minutes per week of moderate physical activity was calculated by summing the moderate and vigorous minutes of activity per week.

A third measure of physical activity collected from all participants by self-report was the Godin leisure time physical activity questionnaire [27-29]. Four questions measure ten-minute episodes of strenuous, moderate and mild leisure time activity, and the number of sweat exercise episodes during a seven-day period. In a previously published validation study among a worker population, the strenuous exercise question was significantly correlated with physiological measures of physical fitness such as VO2max and muscular endurance [29].

### Perceived worksite environment: food choices, physical activity and weight management

Perceptions of the worksite environment regarding food choices, physical activity opportunities and weight management resources were self-reported. Frequency of use of the worksite vending machines and fitness room facilities was queried. Perceived availability of information at the worksite regarding weight control, healthy eating and physical activity was measured. Perceived importance of weight management, healthful food choices and physical activity was self-reported. Five-point Likert response scales were used to assess perceptions and behaviors. For statistical analysis, these were recoded to a dichotomous response variable (eg., agree vs. neutral/disagree; extremely important vs. somewhat/not important).

### Television viewing

Participants self-reported the average number of hours per day spent viewing television and or playing video games. Response options were 0–30 minutes; 31 mins – 1 hr; 1–2 hrs; 2–3 hrs; 3–4 hrs; 4–5 hrs; 5 or more hrs. The number of working televisions present in the household was self-reported.

### Weight concerns

Participants indicated the number of pounds they would have to gain before they noticed the weight gain, and the number of pounds they would have to gain before taking action. Weight concern was defined as the difference between pounds gained before taking action and pounds gained before noticing a weight gain [30,31]. Frequency of self-weighing was self-reported.

### Smoking behavior

Participants self-reported their current and previous smoking behavior. Current smokers were defined as those who answered yes to both of the following questions: "Have you smoked at least 100 cigarettes in your lifetime?" and "Have you smoked a cigarette, even a puff, in the last seven days?"

### Work schedule/hours

Participants reported the average number of hours worked per week. Response options were 0–10 hrs; more than 10 – 19 hours; 20–29 hrs; 30–39 hrs; 40–49 hrs; 50 or more hrs.

### Demographic information

Demographic information was self reported and included age, gender, ethnicity (Hispanic or not), race (coded white vs other for analysis), education completed (high school or less; some college; college or more), annual household income (coded less than $50,000 or $50,000 or more for analysis), and marital status (married vs other for analysis).

### Statistical analysis

Analyses were conducted using SAS Version 8.0 [32]. Means, standard deviations, and frequencies were computed for all variables. Bivariate associations between demographic variables and weight category were examined using Chi square or regression analysis. Multivariate multiple or logistic regression analyses were conducted to examine associations between weight category (normal weight, overweight, obese) and behavioral variables related to energy balance (eg. dietary intake, physical activity, television viewing). Separate equations were used to examine each behavior as a dependent variable and weight category as the independent variable. Covariates included in adjusted analyses were age, gender, education, income, marital status and race. These demographic variables were significantly associated with BMI in the bivariate analyses or were related to BMI in the existing literature. Total energy was included as a covariate in analyses of dietary intake variables to allow for examining nutrient density (nutrient intake independent of energy intake) as a measure of quality of the diet. Adjusted associations were considered significant when p < .05.

## Results

### Demographic and work-related variables

Demographic and work-related variables are shown in Table 1. Seventy-two percent of the employees who completed the surveys were bus drivers; 16% were bus maintenance staff; 7% other jobs (such as dispatchers) and 3% were managers. Below, the total sample is referred to as transit workers for simplicity of presentation. Descriptive statistics on the demographic and behavioral variables are presented first. Associations between body weight category and demographic and behavioral variables follow.

**Table 1:** Demographic Variables By Weight Status Among 1092 Metropolitan Transit Workers

		**Body Mass Index Category**		
	**Total**	**< 25**	**>= 25 < 30**	**>= 30**

**Survey Data**				
**N**	**1092**	**132**	**343**	**617**
Age (years)	47.6 (10.2)	44.6 (11.4)^A^	47.9 (9.8)	48.1 (10.0)***
Gender (male; %)	78.4	74.6	84.9^A^	75.7**
Race (white; %)	65.9	65.1	67.1	65.5***
Education (%)				
high school/vocational	48.9	44.6	48.8	49.8
some college	37.7	39.2	35.2	38.7
college degree or more	13.4	16.1	15.9	11.4
Income (annual household; %)				
less than $55,000	42.5	53.5^A^	41.1	41.0
≥= $55,000	57.5	46.5	58.9	59.0
Marital status (married; %)	60.0	52.7	65.5^A^	58.6*
Smoking status (current smokers;%)	25.4	25.0	26.5	24.3
Years at transit company (%)				
up to three years	5.5	10.0	5.8	4.5***
three to < six years	25.9	30.7	28.9	23.2
six to < fifteen years	38.2	34.5	31.2	42.6
fifteen years or longer	30.3	24.6	33.9	29.4
Hours worked per week	41.6 (8.9)	39.6^A ^(9.4)	41.4 (9.5)	42.2** (8.3)
Weight (kgs)	97.6 (0.7)	68.3^A ^(1.5)	84.0^B ^(0.9)	111.7^C^*** (0.7)
Body mass index (kg/m^s^)	32.3 (7.3)	22.9^A ^(.17)	27.7^B ^(1.4)	36.9^C^*** (6.4)
Scales in home (%)				
none	36.4	42.1	32.5	37.3
One	54.2	51.6	59.4	51.8
two or more	9.5	6.3	8.0	10.9
Self-weighing frequency (%)				
never/once per year	25.8	31.8	24.2	25.4
every few months/monthly	47.1	42.4	46.6	48.5
weekly or more	26.9	25.8	29.2	26.7

Note. Variables are unadjusted means (standard deviations) or percents. p < .05 ** p < .01 *** p < .001

Different superscripts indicate significantly different means or percents.

Seventy-eight percent of the employees were men, with an average age of 47 years (age range 19 – 79 years). Sixty-two percent were white. Forty-eight percent had completed high school/vocational school or had less education; and 42% reported annual household incomes before taxes of less than $50,000. Most workers had been employed with the transit company six or more years; about one-third had been working with the transit company 15 years or longer.

### Body weight, food choices and dietary intake

Overall, the prevalence of obesity among the transit workers was very high (Table 1). The average BMI was 32.3 kg/m^2^. Eighty-seven percent were overweight or obese (BMI >= 25 kg/m^2^) and 56% were obese (BMI >= 30 kg/m^2^).

Food choices from the food frequency survey and dietary intake variables from the 24-hour recall substudy are shown in Table 2. According to the food frequency survey (Table 2, top panel), on average during the past month, 55% of drivers reported eating fruit 3 or more times per week (34% reported 5–7 times per week); 58% consumed vegetables other than salad or french fries three or more times per week (30% reported 5–7 times per week);, and 14% consumed french fries three or more times per week (4% reported 5–7 times per week). Thirty-four percent reported consumption of sugar-sweetened soft drinks three or more times per week (22% reported 5–7 times per week). Sixty-five percent reported consuming food from fast food restaurants less than once per week during the past month. Frequency of reported consumption of sweets and snack foods such as chips, ice cream, pastry, cookies and candy was low. Reported use of the garage vending machines during the past month was modest. Thirty-one percent reported using the snack food vending machine three or more times per week during the past month (16% reported 5–7 days per week); and 34% reported using the cold beverage vending machine three or more times per week (19% reported 5–7 days per week). Only 8% reported using the cold food vending machine three or more times per week (3% reported 5–7 days per week).

**Table 2:** Food Choices By Weight Status Among 1092 Metropolitan Transit Workers

		**Body Mass Index Category**		
	**Total**	**< 25**	**>= 25 < 30**	**>= 30**

**Survey Data**				
**N**	**1092**	**132**	**343**	**617**
Food Frequency (m (se) frequency – quantity sum across items)				
Fruit Vegetable (4 items) %	15.2 (.35)	14.1 (1.01)	15.8 (.62)	15.1 (.47)
Snack Sweets (9 items) &	21.6 (.72)	19.2 (2.09)	19.7 (1.29)	22.9 (.96)
Sweetened Beverages (2 items) $	48.1 (1.65)	41.4 (4.78)	42.5 (2.94)	52.6^A^** (2.21)
Fast Food Restaurant (m(se)) (times per week during past month)	1.14 (.05)	.97 (.13)	1.12 (.08)	1.18 (.06)
Garage Vending Machines (past month frequency of use; 3 or more times per week; %)				
Snack food	31.7	24.9	25.9	37.0**
Cold food	8.3	4.9	5.9	10.4**
Hot beverage	32.0	32.6	31.6	31.8
Cold beverage	33.6	26.8	25.8	39.5**
Dietary Recall Data
**N**	**160**	**19**	**45**	**96**
(m (se))				
Energy (kcal/d)	2359 (76)	2736 (219)	2289 (143)	2291 (99)
Fat (%kcal/d)	35.5 (.69)	36.9 (1.9)	31.8 (1.3)^A^	36.4 (.9)**
Saturated Fat (%kcal/d)	12.2 (.34)	13.1 (1.0)	10.8 (.6)	12.4 (.4)
Fruit (svg/d)#	1.6 (.16)	1.6 (.48)	2.0 (.31)	1.5 (.22)
Vegetables/no potatoes#	2.5 (.24)	1.9 (.70)	2.6 (.45)	2.6 (.31)
Meets Fruit/Vegetable Recommendation 5 Servings/Day (%)	42.9	33.5	42.1	45.3
Alcohol (svg/d)#	.45 (.11)	.20 (.33)	.77 (.21)	.34 (.15)
Milk, High fat (svg/d)#	.50 (.07)	1.09^A ^(.20)	.28 (.13)	.48 (.09)**
Milk, Low fat (svg/d)#	.46 (.08)	.12 (.24)	.37 (.15)	.57 (.10)
Snack foods (svg/d)#	.83 (.10)	.82 (.28)	.81 (.18)	.84 (.13)
Sweet foods (svg/d)#	1.7 (.18)	1.5 (.53)	1.7 (.34)	1.7 (.24)
Sugar Sweetened Beverages# (svg/d)	1.1 (.12)	1.6 (.36)	1.1 (.23)	.91 (.16)

Note. Means adjusted for age, gender, race, income, education and marital status.

***p < .001 **p < .01 *p < .05 Different superscripts indicate significantly different means by post hoc comparison p < .05.

m = mean. se = standard error.

#Adjusted for above variables plus total energy.

Svg = servings. d = day.

% Fruit, fruit juice, salad, fried potatoes.

& Chips, popcorn, pretzels, ice cream, cookies, pastry, muffins, chocolate candy, other candy.

$ Sugar sweetened soft drinks, fruit drinks

Results from the 24-hour dietary recall substudy are shown in the bottom panel of Table 2. Consistent with the food frequency survey, transit workers reported an average of 1.6 fruit servings and 2.5 vegetable servings per day, and 42% met the five or more servings per day national recommendations for fruit and vegetable intake. However, dietary fat intake was higher than desired (35%), and average sweets and snack food intake was 1.7 servings and 0.8 servings per day. Average consumption of sugar sweetened beverages was one serving per day.

### Physical activity and sedentary behaviors

Sedentary and physical activity behaviors are shown in Table 3. Self-report data from the survey is shown in the top panel and data from the objective accelerometry substudy are shown in the bottom panel. Self-reported physical activity was much higher than expected, based on answers to the modified IPAQ. Workers reported on average walking a total of 93 minutes per day. In addition to time spent walking, workers reported on average a total of 60 minutes of vigorous activity and 75 minutes of moderate activity per day. Eighty-five percent of the employees met national recommendations for physical activity of 150 minutes of moderate activity per week. Consistent with the results from the IPAQ, self-reported frequency of strenuous, moderate and mild physical activity episodes based on responses to the Godin leisure time physical activity questionnaire showed high levels of physical activity. On average per week, about eight episodes of physical activity of ten minutes or longer were reported; including two strenuous episodes (see Table 3). Sedentary behavior levels also were high. Transit workers reported viewing about 2 hours of television per day and sitting a total of 9 hours per day.

**Table 3:** Physical Activity By Weight Status Among 1092 Metropolitan Transit Workers

		**Body Mass Index Category**		
	**Total**	**< 25**	**>= 25 < 30**	**>= 30**

**Survey Data**				
**N**	**1092**	**132**	**343**	**617**
International Physical				
Activity Questionnaire				
(m (se))				
Walking (mins/d)	93.3 (2.4)	103.4 (6.9)	97.4 (4.3)	88.4 (3.2)
Vigorous Activity (mins/d)	59.5 (2.1)	63.0 (6.1)^A^	65.8 (3.8)^AB^	55.3 (2.8)^B^
Moderate Activity (mins/d)	75.0 (2.2)	79.8 (6.5)	85.2 (4.0)	68.4 (3.0)
Meets Physical Activity Recommendations (150 mins/d; %)	85.1	88.6^AB^	87.4^A^	82.8^B^**
Sitting (hours/d)	9.3 (.13)	8.6 (.36)	8.6 (.22)	9.8 (.17)^A^**
Godin Leisure Time Physical Activity				
(10-min episodes per week)				
(m (se))				
Strenuous	1.9	2.2	2.3	1.6
	(.09)	(.25)	(.16)	(.12)^A^**
Moderate	2.6	3.0	2.8	2.5
	(.11)	(.33)	(.20)	(.15)
Mild	3.5 (.14)	3.7 (.40)	3.5 (.25)	3.4 (.18)
Sweat frequency (%)				
Never	24.1	24.6	23.3	24.5
Sometimes	55.1	54.1	50.2	58.0
Often	20.8	21.3^AB^	26.5^A^	17.5^B^**
Television Viewing (hours/d)	2.0 (.04)	1.6 (.11)	1.8 (.07)	2.2 (.05)^A^**
Televisions in Home (>= 4 sets; %)	31.8	23.4^A^	30.4^AB^	34.0^B**^
Fitness Room Use				
(past year; >= 1 time;%) Garage Fitness Room	29.3	27.2	28.7	30.2
Non-worksite Fitness Room	49.7	43.7	53.3	49.0
Exercise on Driving Route (past year; >= 1 time;%)	46.4	47.2	53.9	42.3
**Accelerometry**				
**N**	**158**	**28**	**36**	**94**
(mins/d)(m (se))				
Moderate/Vigorous	16.7 (1.2)	24.4 (2.9)^A^	20.0 (2.5)^AB^	15.1 (0.5)^B^**
Light	232.5 (5.3)	255.5 (13.1)	248.8 (11.3)	220.0 (6.7)^A^*
Inactive	342.8 (6.2)	323.1 (15.7)	337.3 (13.5)	343.8 (8.1)

Note. Means adjusted for age, gender, race, income, education and marital status. m = mean. se = standard error. mins = minutes. d = day. **p < .01 *p < .05. Different superscripts indicate significantly different means, post hoc comparison p < .05.

By contrast with the high self-reports of physical activity, 83% of the transit workers reported never using the fitness room at work during the past month, 60% never used a fitness room at a non-work location during the past month, and 63% of the drivers never exercised during the driving route during the past month.

Physical activity levels indicated by the Actigraph data were much lower than the self-reported physical activity levels. On average drivers were moderately or vigorously active for 16 minutes per day. Almost six hours per day were spent inactive, and almost four hours were spent in light activity.

### Perceived worksite environment and individual attitudes about healthy food choices, physical activity and weight management

Perceived worksite food, physical activity and weight management environmental variables and individual perceptions are shown in Table 4. Overall, the worksite was not perceived as very supportive of healthful food choices, physical activity and weight management. Fifty-two percent of the drivers agreed that it was hard to get fruits and vegetables at work, and 62% reported that they found it hard to be physically active at work. Only 32% agreed that there was a lot of information available at work on healthful eating, and 34% agreed that there was a lot of information available at work about physical activity. Perceived social support for healthful food choices, physical activity and weight management behavior was modest. Levels of reported social support were similar across the three behaviors (food, physical activity, weight management) (Table 4). Across all three behaviors, the highest perceived support was from family members. However, perceived support from family was rated only at the scale midpoint, and friends and co-workers support were rated below the scale midpoint.

**Table 4:** Perceived Work Environment and Attitudes About Healthy Eating and Physical Activity Among 1092 Metropolitan Transit Workers

		**Body Mass Index Category**		
	**Total**	**<25**	**>= 25 < 30**	**>= 30**

**Survey Data**				
**N**	**1092**	**132**	**343**	**617**
**Perceived access at work** (hard to get at work; agree; %)				
Fruit/vegetables	51.9	48.9	52.9	51.9
Physical activity	61.7	52.7	56.7	66.3^A^**
**Perceived information at work** (lots of information at work: agree; %)				
Healthy eating	32.6	33.2	34.5	31.4
Physical activity	33.7	35.9	36.3	31.7
Weight Management	26.6	28.9	29.0	24.7
**Perceived easy to do at workplace **(agree: %)				
Healthy eating	15.8	17.8	18.5	13.8
Physical activity	30.3	33.2^AB^	36.8^B^	25.9^A^**
Weight management	18.5	27.5	24.9	13.0^A^***
**Perceived social support**				
(1 = not at all supportive; 5 = very supportive) (m,se)				
Healthy Food Choices				
Family	3.1(.05)	2.7 (.15)^A^	3.1 (.09)	3.2 (.07)**
Friend	2.3 (.05)	2.0 (.15)	2.4 (.09)	2.3 (.07)
Co-worker	1.6 (.05)	1.5 (.14)	1.6 (.08)	1.6 (.06)
Physical Activity				
Family	3.0 (.05)	2.8 (.15)	3.0 (.09)	3.0 (.07)
Friend	2.3 (.05)	2.1 (.15)	2.3 (.09)	2.3 (.07)
Co-worker	1.5 (.05)	1.3 (.13)	1.6 (.08)	1.6 (.06)
Weight Management				
Family	2.8 (.05)	2.2 (.16)^A^	2.6 (.10)^B^	3.0 (.07)^C^***
Friend	2.0 (.05)	1.5 (.15)^A^	2.0 (.09)	2.1 (.07)**
Co-worker	1.4 (.04)	1.1 (.13)	1.3 (.08)	1.4 (.06)
**Perceived Importance for Health **(agree; %)				
Food choices	83.7	88.1	82.9	83.2
Physical activity	90.6	93.0	91.0	89.8
Weight management	80.8	67.7^A^	80.9	83.5***
**Perceived Personal Importance for Health **(agree; %)				
Eat fewer calories	44.5	26.6^A^	40.6^B^	50.4^C^***
Be more physically active	67.9	67.8	72.9	65.2
Manage weight	65.5	49.0^A^	65.6	69.0***
**Perceived Weight Concerns **(m; se)				
Pounds gain to notice	10.3 (.44)	9.4 (1.3)	10.4 (.79)	10.5 (.60)
Pounds gain to take action	16.4 (.73)	18.7 (2.1)	15.4 (1.3)	16.7 (1.0)
Weight concern (notice – take action)	-6.3 (.59)	-9.4 (1.7)	-4.9 (1.0)	-6.5 (.82)

Note. Means adjusted for age, gender, race, income, education and marital status. m = mean. se = standard error. ***p < .001 **p < .01 *p < .05 Different superscripts indicate significantly different means by post hoc comparison p < .05.

Sixty-five percent of the drivers reported that weight control was personally important; 68% reported that physical activity was personally important; but only 44% reported that eating fewer calories was personally important.

Frequency and intensity of weight control behaviors was modest in the transit worker population. Two-thirds of the transit workers reported the presence of a scale in their home. On average, employees reported self-weighing between every month and every couple of months. A general measure of weight concern, the difference between the amount of weight gain to notice versus to act on was 6.0 lbs. Although transit workers reported noticing a weight gain of 10 lbs, they were not motivated to take action until having gained 16 lbs.

### Associations between body weight, eating and physical activity behaviors

Few differences in food choices and physical activity behaviors were observed among transit workers of different body weight categories. According to the dietary recall measure (Table 2), total energy intake did not significantly differ among drivers by body weight category. However, overweight workers reported lower percent fat energy compared to the normal weight or obese workers. Intake of fruit, vegetables, sweets, and snack foods did not differ among employees by body weight category. Obese employees reported more frequent use of the snack, cold food and cold beverage vending machines compared to the other employees (Table 2).

By contrast, differences were observed among transit workers of different body weight categories for the physical activity and sedentary behavior variables (Table 3). Based on responses to the IPAQ questionnaire, obese workers reported significantly less moderate physical activity, and significantly more time sitting, compared to other drivers. Compared to overweight employees, obese employees were significantly less likely to meet the recommendation for 150 minutes per week of moderate physical activity. Results from responses to the Godin questionnaire were consistent with the IPAQ and showed that compared to normal weight or overweight transit workers, obese transit workers reported significantly fewer episodes per week of strenuous physical activity. Results from the Actigraph objective physical activity measure showed that obese workers spent significantly less time in moderate or vigorous activity, or in light activity, compared with other workers.

Obese transit workers reported watching significantly more hours of television per day, and reported a significantly greater number of television sets present in their household. Compared to normal weight and overweight workers, obese workers were more likely to agree that it is hard to be physically active at work, and that weight management and eating fewer calories were personally important.

Frequency of self-weighing did not differ among transit workers by body weight (Table 4), nor did transit workers of different body weight categories differ in the amount of weight they needed to gain in order to notice that they had gained weight, or in order to take action to prevent further weight gain.

## Discussion

The results of the present study show that compared to US national data, the prevalence of obesity and overweight in this metropolitan transit worker population is extremely high. The prevalence of obesity was 56%, compared to 35% among US adults [2]. These findings are of concern because of the potential serious health risks that such high levels of obesity present to individuals and to the employer in terms of health care costs and economic costs from lost workdays due to illness and disability [5,7,33-36].

Food choices and physical activity behaviors reported by transit workers seem to point to generally healthful patterns, which raises the question of why are these workers obese? Dietary intake did not appear to differ significantly from the average US adult population. Frequency of fruit and vegetable intake and intake of snack foods, sweets and fast food was typical of US adults more generally [37-40]. Mean energy intake, percent fat energy, fruit and vegetable intake among the transit workers was comparable to recent national data (37–40) (mean energy: 2359 kcal/day versus 2363 kcal/day, respectively; 35.5% fat versus 33.7% fat; 1.6 fruit servings/day versus 1.5 servings/day; 3.4 vegetable servings/day versus 4.1 servings/day). Television viewing hours were also similar to the amounts reported in US adult populations [8,30]. The lack of unusual reported dietary intake and food choices among the transit workers, who are severely overweight as a group, is possibly due to social desirability in reporting [41-43]. Generally, people tend to under-report their intake, and greater under-reporting has been documented among the overweight [41,43]. In the present study, participation rates in the dietary recall substudy did not differ by gender, body mass index, or age, so selection bias along variables related to dietary intake and physical activity appears to be unlikely in the substudy samples. Those who participated in the substudies could have been more health conscious in general. However, comparison of those who participated in the substudies with those who did not showed no differences on measures of the perceived personal importance of healthy eating, physical activity and weight management (data not shown). Although these food measurements may not provide valid information on absolute levels of dietary intake among severely overweight populations such as those in the present study, they may capture intervention-related change if biased reporting does not differ by treatment group assignment.

Absolute levels of self-reported walking, moderate and vigorous physical activity were unrealistically high. Over-reporting of absolute physical activity levels has been reported in population-based studies that used the IPAQ [24-26]. For example, mean vigorous activity was 40 minutes/day in Rutten et al [24] and 30 minutes per day in Rzewnicki et al [26], compared with 59 minutes/day in the present study. Walking minutes (mean) reported were 85 minutes/day in Rutten et al [24] and 74 minutes/day in Rzewnicki et al [26], compared with 93 minutes/day in the present study. The reason for the higher activity levels more generally observed with the IPAQ could be due to the inclusion of a greater number of activity domains (eg. home chores, transportation-related physical activity, work activity) compared with typical physical activity questionnaires that focus only on leisure time physical activity. Similar to a food frequency questionnaire, simply including a greater number of questions to assess the physical activity domains may lead to inflated reports compared to measures with fewer items. In addition, the modified format of the IPAQ that was used in the present study might have contributed to the reported higher physical activity levels. Instructions and response categories were modified based on our pilot study with over 200 bus operators at a pilot garage prior to data collection for the main study. Based on the high absolute level of activity reported for each of the activity categories (light, moderate and vigorous), it is hypothesized that drivers may have reported activity amounts for the past week (sum of seven days), rather than a daily average. Even if this were the case, levels of reported physical activity seem high. Socially desirable responding may have contributed to the overreporting observed here relative to the very low physical activity levels measured with accelerometry. In the present study, correlations between the accelerometer and the self-report physical activity measures were low. For example, the correlation between the moderate and vigorous accelerometer variable and the vigorous IPAQ variable was r = .19 (p < .02). Stronger correlations were observed between the Godin strenuous variable and the accelerometer strenuous variable (r = .26; p < .001). Clearly, more research is needed to determine the most valid self-report measure for estimating intervention-related changes in physical activity in bus operators or other employee populations.

Transit workers in the present study appeared to be aware of the importance of weight management, healthful food choices and physical activity behavior. There was an apparent slight preference among bus drivers for physical activity versus calorie reduction for weight management. However, the clear majority reported that is was difficult to be physically active at work, and few reported using the garage fitness facilities or exercising on the bus route. Most drivers did not agree that there was a lot of information at work on physical activity, healthy eating or weight management.

Few differences were observed in dietary intake and physical activity behaviors by transit workers' overweight/obese status. However, those that were observed are consistent with the literature on behaviors that are probable contributors to obesity [7,8,31]. Total energy intake, based on the dietary recalls, did not differ by weight category, perhaps due to social desirability bias in reporting. Obese transit workers reported more frequent intake of regular soft drinks, greater use of the cold beverage vending machines, more hours sitting, more hours viewing television, and less strenuous, moderate and light physical activity, compared to other transit workers. Obese workers also reported greater difficulty being physically active and eating healthfully at work, and reported greater importance of weight management, calorie reduction and physical activity.

## Conclusion

The findings suggest that obesity prevention interventions that target transit workers through garage worksites should take advantage of the existing interest among bus drivers for increasing regular physical activity as a weight management strategy. Strategies designed to increase the use of garage fitness facilities, and that incorporate exercise into the driving route, may be useful intervention approaches that are currently under-utilized. Based on the greater usage reported by obese drivers of the garage beverage vending machines, attention to making available only low-energy beverages might be considered. Greater television viewing among obese drivers also suggests that limiting television access at the garage might be warranted. Drivers appear to be aware of the importance of healthful eating, physical activity and weight management. Given the high awareness and motivation, the provision of more information at the worksite, combined with environmental changes to make healthful eating and physical activity more attractive and accessible, could be an important intervention approach to prevent obesity in this employee population.

## Competing interests

The author(s) declare that they have no competing interests.

## Authors' contributions

All authors contributed to the design of the study. SAF wrote the manuscript. LJH, TLT, and PJH helped write the manuscript. PJH provided guidance with the statistical analysis of the data.
